# Comparison of the Outcome of Low Dose and High-Dose Corticosteroid in the Treatment of Idiopathic Granulomatous Mastitis

**DOI:** 10.31557/APJCP.2020.21.4.993

**Published:** 2020-04

**Authors:** Majid Montazer, Maryam Dadashzadeh, Seyed Ehsan Mousavi Toomatari

**Affiliations:** *Department of General Surgery, Imam Reza Hospital, Faculty of Medicine, Tabriz University of Medical Sciences, Tabriz, Iran. *

**Keywords:** Idiopathic granulomatous mastitis, prednisolone, therapy

## Abstract

**Background:**

Idiopathic granulomatous mastitis (IGM) is a rare chronic inflammatory breast condition with unknown etiology. Different treatments including corticosteroids have been recommended with no universal consensus. In this study we evaluated the efficacy of low dose vs. high dose prednisolone in treatment of IGM.

**Methods:**

In this randomized clinical trial, 30 female patients with IGM were randomly allocated to receive low dose or high dose prednisolone. First group received 5 mg daily prednisolone, while the second group received 50 mg for three days, 25 mg for the next three days and then 12.5 mg for further three days and 5 mg daily afterwards, both for two months. Patients were evaluated 2, 3, 6 and 12 months after treatment. The success and recurrence rate was compared between groups.

**Results:**

High dose group had significantly higher rate of remission compared to low dose group (93.3% vs. 53.3%, p=0.03). One patient in high dose group and 5 patients in low dose group underwent lumpectomy due to persistent symptoms. Two other patients in low dose group received high dose treatment after three months due to no change in symptoms. Among patients with remission, recurrence was also significantly lower in high dose compared to low dose prednisolone (0% vs. 37.5%, p=0.04).

**Conclusion:**

High dose prednisolone has high success rate with lower recurrence in the treatment of IGM and could reduce the need for surgery. However, further studies are necessary to confirm these findings.

## Introduction

Idiopathic granulomatous mastitis (IGM) is a rare, benign and chronic inflammatory breast condition (Akcan et al., 2014; Salehi et al., 2017; Yukawa et al., 2015). Its etiology is unknown, but association with immunologic and connective tissue diseases, mycobacterium and fungus infections or any hypersensitivity reactions of the breast lobules are considered as possible cause (Akcan et al., 2014; Korkut et al., 2015; Salehi et al., 2017). IGM can mimic breast cancer (Akcan et al., 2014; Karanlik et al., 2014), so it is necessary to diagnose the disease to prevent unnecessary interventions. 

IGM is diagnosed histopathology with the presence of non-caseified granulomatous inflammation. There is not a pathogno¬monic sign on ultrasonography, mammography and magnetic resonance imaging. It usually occurs in women in the childbearing ages with most common period 2-6 years after pregnancy (Korkut et al., 2015). IGM seems to be related to the ethnicity with highest reported cases from Asia and Mediterranean region (Chirappapha et al., 2018). 

On the other hand, due to lack of a definitive treatment plan, complications of empiric treatment, such as allergic reaction to antibiotics and poor cosmetic procedures result in following repeated surgical interventions which threaten the patients (Garcia-Rodiguez and Pattullo, 2013; Gurleyik et al., 2012). In fact, the etiology of IGM is unknown. IGM is characterized pathologically by the presence of chronic granulomatous lobulitis in the absence of an obvious etiology. Although the frequency of IGM is increasing fast, only a few hundred cases have been reported worldwide (Akcan et al., 2014).

The management strategy for IGM is controversial. Use of corticosteroids alone or in combination with methotrexate or azathioprine and antibiotics especially azithromycin, abscess drainage, and wide local excision of the lesions have been reported for the treatment of IGM (Akcan et al., 2014; Atak et al., 2015; Salehi et al., 2017; Sheybani et al., 2015; Yaghan et al., 2019; Yukawa et al., 2015). Considering the efficacy of the corticosteroids in the previous studies which are mostly retrospective, there is a need for clinical trials to confirm these findings. In this study we aim to evaluate the efficacy of low dose vs, high dose prednisolone in the treatment of IGM. 

## Materials and Methods

In this randomized clinical trial, 30 female patients with pathologically confirmed IGM visiting Imam Reza Hospital, Tabriz between 2017 and 2019 were recruited. Patients with other types of granulomatous or immunologic diseases, chronic mastitis, history of breast surgery, pregnant or breastfeeding, use of corticosteroids in the last three months or with any contraindications to use corticosteroids were excluded. The ethics committee of Tabriz University of Medical Sciences approved the study protocol and informed consent were obtained from all patients. 

The study sample size for each group was calculated 15 cases considering an effect size of d ≥0.60 as statistically significant in a two-tailed test with α = 0.05 and power of 0.80. 

Patients were randomly allocated to two groups (Figure 1); Group I received low dose prednisolone (5 mg daily) and group II received high dose prednisolone (50 mg for three days, then 25 mg for the next three days, 12.5 mg for three days and 5 mg daily afterwards) bot for two months. No further prednisolone was administered after the end of the trial. Patients were evaluated for improvement in symptoms before, 2, 3 and 6 months after treatment. 

Successful treatment was considered as remission of the symptoms and size of the lesion after treatment. Recurrence was recorded in those patients with fully remission with corticosteroid use. 

*Statistical analysis*

All data were analyzed using SPSS20. Results are expressed as mean ± SD or percentage. Chi square test, Fischer’s exact test or independent T-test were used to compare data between groups. p values of less than 0.05 were considered statistically significant. 

## Results

Thirty female patients with IGM were evaluated. Breast involvement was unilateral in all cases (11 in left and 19 in right side). All women were in their reproductive age and had at least two children. Serologic and bacterial studies were all negative. However, prophylactic antibiotic therapy was initiated for all participants. 

Histologic and pathologic samples were acquired by core needle biopsy in 13, FNA in 8 and open biopsy in 8 women. One patient had first FNA showing multiple abscess and received antibiotic therapy, but had no response and then core needle biopsy was performed. All samples were diagnostic for IGM. 

Table 1 demonstrates baseline and follow-up findings between two different groups. Two groups are similar regarding age, months since last breastfeed and side of the lesion. The most common presenting symptoms were painful mass in both groups. Patients were followed for more than 12 months. There was no side effects regarding use of prednisolone. 

Patients receiving high dose prednisolone had significantly higher rate of remission compared to low dose group. In high dose group, four patients improved in the first two months, 4 patients after three months and 3 patients after six months. One patient underwent lumpectomy after 6 months due to the resistance to treatment. The remaining three patients had fully improvement at the end of 12 month follow-up. The 8 patients with successful treatment in low dose group showed improvement in two months (4 cases) 3 months (2 cases) and 6 months (2 cases) after treatment. Two patients after third month and 3 patients after sixth month had progress to abscess and fistula and after treatment with proper antibiotics, underwent lumpectomy. Two other patients after no change in outcome in the third month, received high dose prednisolone and were fully cured. 

Of the successful treatment in each groups, recurrence was observed in three patients in low dose group with significant difference between groups (p=0.04). All these three cases responded to treatment in the first two months, but had recurrence in the sixth month. All three was treated with high dose prednisolone and had no recurrence afterwards.

**Table 1:** Baseline and Follow-up Findings between Two Different Groups

		Low dose	High dose	*P*-value
Age (years)		34.80±9.31	35.60±7.64	0.79
Month since last breastfeed		42.06±21.83	45.40±22.92	0.68
Breast involved	Left	7 (46.7%)	4 (26.7%)	0.26
	Right	8 (53.3%)	11 (73.3%)	
Presenting symptom	Painful mass	10 (66.7%)	7 (46.7%)	------
	Pain and erythema	4 (26.7%)	5 (33.3%)	
	Fistula with drainage	1 (6.7%)	3 (20%)	
Remission		8 (53.3%)	14 (93.3%)	0.03*
Remission time (Months)		3.25±1.75	5.28±3.93	0.49
Recurrence		3 (37.5%)	0%	0.04*
Follow-up (months)		12.60±2.19	13.13±1.99	0.18

* p is two sided significant.

**Diagram 1:**
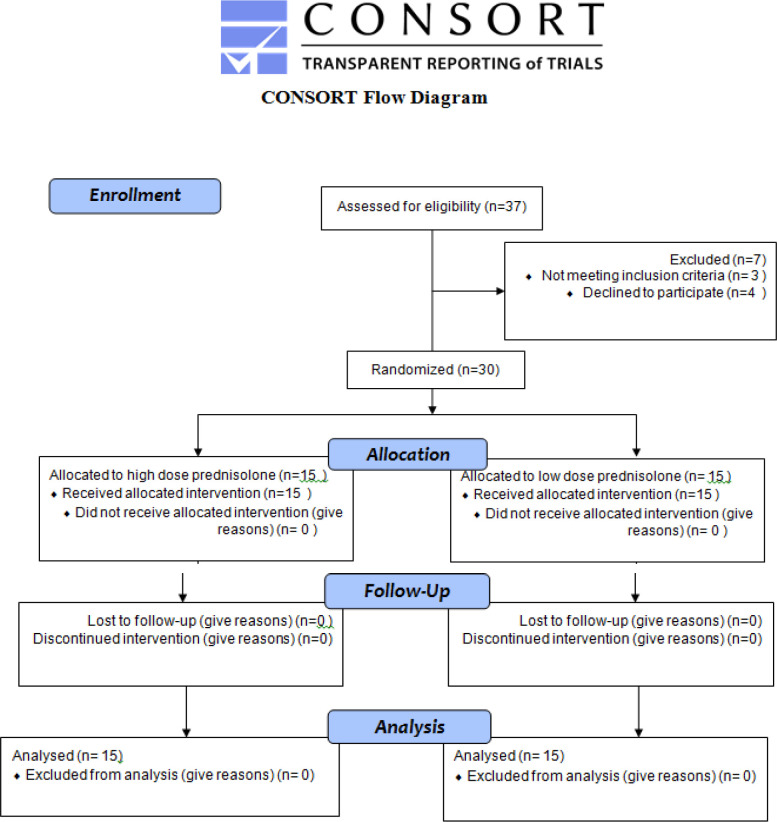
CONSORT Flow Diagram

## Discussion

In this study we evaluated the efficacy of low dose vs. high dose prednisolone in treatment of IGM and observed that both low dose and high dose could be useful in the treatment of the disease with higher success rate for the high dose group. There was also low rate of remission for high dose group. 

Different treatments have been recommended for IGM during the last 40 years. One of the first studies recommended that corticosteroids would be appropriate treatment for IGM (DeHertogh et al., 1980). Corticosteroids are usually used alone or in combination with other drugs or surgery. Uysal et al., (2018) in their major retrospective study of 720 IGM from 22 centers in Turkey reported using corticosteroids in 39% of cases. Sakurai et al., (2011) reported success rate of 87% in patients receiving corticosteroids. Karanlik et al., (2014) observed complete response after two months treatment with low-dose prednisolone. The response rate (53.3%) in our patients treated with low dose prednisolone was lower than previous studies. Su et al., (2005) also observed that administering high dose prednisolone could successfully treat the patient with IGM with no relapses, as observed in our study. Erozgen et al., (2010) also reported that using corticosteroids in non-complicated cases of IGM is very efficient. 

Besides the efficiency of a treatment, the relapse rate is important after treatment. We observed no recurrences in the high dose prednisolone, while low dose group had 37.5% rate of recurrence. Our subjects were followed for almost 12-13 months. The overall recurrence rate reported by Uysal et al., (2018) was 17%. Unlike our findings, Azlina et al., (2003) reported a recurrence rate of 50% after treatment with high dose prednisolone. Similarly, Akbulut et al., (2011) in their retrospective study of 541 IGM cases, reported high recurrence rate in patients receiving corticosteroids. The differences in these findings could be due to the follow-up duration, which was lower in our patients. 

If there are complications such as persistent abscess or fistula, surgery seems to be a better treatment option. In our patients, one patient in high dose and 5 patients in low-dose prednisolone underwent lumpectomy due to persistent pain and/or abscess progressed to fistula. Erozgen et al., (2010) also indicated that surgery is preferred method after relapse, abscess or fistula.

In our study low dose or high dose prednisolone had no drug related side effects; however, in the literature there are concerns about these side effects including glucose intolerance and cushingoid features (Ayeva-Derman et al., 1999; Belaabidia et al., 2002).

There are no known standard treatment for IGM; however, studies have recommended to use surgery following a period of corticosteroid therapy which would be the treatment of choice in the future. This method accompanied with no significant rate of relapse after longtime follow-up (Akcan et al., 2014). Whether high dose prednisolone alone or in combination with other treatments including surgery are better treatments need further randomized studies.

In conclusion, high dose prednisolone has high success rate with lower recurrence in the treatment of IGM and could reduce the need for surgery. However, further studies are necessary to confirm these findings. 

## Limitations

Although being a clinical trial was the strength of this study, it had some limitations; the first limitations was the low sample size of the study population; as another limitation, we did not assess disease remission with sampling which is painful and not accepted by the patients and only sonography and physical examination was used. Another limitation would be that we did not compare our findings with other treatments including surgery, mostly because there are no gold standard treatment for IGM. 
